# Altered Regional Brain Activity Underlying the Higher Postoperative Analgesic Requirements in Abstinent Smokers: A Prospective Cohort Study

**DOI:** 10.1523/JNEUROSCI.0109-25.2025

**Published:** 2025-12-08

**Authors:** Kai Wei, Kunming Tao, Yanzhi Bi, Xuerong Miao, Hongmei Xiao, Yue Zhang, Haibo Qiu, Jiao Zhu, Qianbo Chen, Ling Shen, Huihong Xu, Min Ma, Li Hu, Kui Wang, Zhijie Lu

**Affiliations:** ^1^Department of Anesthesiology, Shanghai Eastern Hepatobiliary Surgery Hospital, Naval Medical University, Shanghai 200438, China; ^2^State Key Laboratory of Cognitive Science and Mental Health, Institute of Psychology, Chinese Academy of Sciences, Beijing 100101, China; ^3^Department of Psychology, University of Chinese Academy of Sciences, Beijing 100049, China; ^4^Department of Hepatic Surgery II, the Eastern Hepatobiliary Surgery Hospital, Naval Medical University, Shanghai 200438, China; ^5^Department of Anesthesiology, Minhang Hospital, Fudan University, Shanghai 201100, China

**Keywords:** abstinent smokers, nicotine withdrawal-induced hyperalgesia, postoperative analgesic requirements, regional brain activity, resting-state functional magnetic resonance imaging

## Abstract

Perioperative abstinent smokers experience heightened pain sensitivity and increased postoperative analgesic requirements, likely due to nicotine withdrawal-induced hyperalgesia. However, the underlying neural mechanisms in humans remain unclear. To address this issue, this study enrolled 60 male patients (30 abstinent smokers and 30 nonsmokers) undergoing partial hepatectomy, collecting clinical data, smoking history, pain-related measures, and resting-state functional magnetic resonance imaging. Compared with nonsmokers, abstinent smokers showed lower pain threshold and higher postoperative analgesic requirements. Neuroimaging revealed altered brain function in abstinent smokers, including reduced fractional amplitude of low-frequency fluctuations (0.01–0.1 Hz) in the ventromedial prefrontal cortex (vmPFC), increased regional homogeneity in the left middle occipital gyrus, and decreased functional connectivity between the vmPFC to both the bilateral middle temporal gyrus and precuneus. Preoperative pain threshold was positively correlated with abstinence duration and specific regional brain activities and connectivity. Furthermore, the observed association between abstinent time and pain threshold was mediated by the calcarine and posterior cingulate cortex activity. The dysfunction in vmPFC and the left anterior cingulate cortex was totally mediated by the association between withdrawal symptoms and postoperative analgesic requirements. These findings suggest that nicotine withdrawal might alter brain functional activity and contribute to hyperalgesia for the abstinent smokers. This study provided novel insights into the supraspinal neurobiological mechanisms underlying nicotine withdrawal-induced hyperalgesia and potential therapeutic targets for postoperative pain in abstinent smokers.

## Significance Statement

Abstinent smokers experienced heightened pain and require more analgesics after surgery, yet the underlying neural mechanisms remain poorly understood. This prospective cohort study identified altered regional brain activity associated with reduced pain thresholds and increased postoperative analgesic requirements in abstinent smokers. We found specific brain regions that were functionally altered and correlated with pain-related outcomes, which mediated the relationship between abstinence and pain-related behaviors. These findings provided novel insights into the supraspinal mechanisms of nicotine withdrawal-induced hyperalgesia and point to potential therapeutic targets for improving postoperative pain management in abstinent smokers.

## Introduction

Approximately 1.18 billion people regularly smoke tobacco (Dai et al., 2022), and the prevalence of smoking prior to surgery exceeds 20% (Howard et al., 2021). Nicotine, the main addictive substance in tobacco, has been shown to induce hyperalgesia during withdrawal (Ditre et al., 2018; LaRowe and Ditre, 2020). Surgical patients are typically asked to quit smoking due to the elevated risk of postoperative complications associated with tobacco use, including delayed wound healing, tissue infections, respiratory dysfunction, and/or sepsis (Aspera-Werz et al., 2022). Numerous clinical studies have reported that abstinent smokers experience greater postoperative pain and require higher doses of analgesics compared with nonsmokers (Weingarten et al., 2011; Chiang et al., 2016; Shen et al., 2017; Iida et al., 2022).

Nicotine withdrawal-induced hyperalgesia may be caused by changes in the nervous system. Several animal studies have examined the spinal mechanisms underlying this phenomenon. For example, Lina et al. demonstrated that interferon regulatory factor 8 plays a crucial role in nicotine withdrawal-induced hyperalgesia by enhancing microglia activation and the expression of spinal P2X purinoceptor 4 and brain-derived neurotrophic factor in mice (Guo et al., 2020). Zhang et al. found that nicotine withdrawal alters basal levels of glutamate decarboxylase 67 and 65, opioid receptors, endorphins, and γ-aminobutyric acid, contributing to heightened pain sensitivity in an animal model (Zhang et al., 2019). Although animal studies (including our work in raphe nuclei) implicate supraspinal mechanisms in nicotine withdrawal-induced hyperalgesia (Schmidt et al., 2001; Shen et al., 2021), direct neuroimaging evidence in humans remains scarce.

Over the past decade, resting-state functional magnetic resonance imaging (rs-fMRI) has been widely used to investigate the brain dysfunction related to nicotine dependence and cessation (Beaver et al., 2011; Lesage et al., 2020). A recent meta-analysis which encompassed a total of 35 rs-fMRI studies revealed that individuals with tobacco use disorder exhibited increased intrinsic function in the right cerebellum crus2, left superior frontal gyrus, left inferior parietal gyrus, and left supplementary motor area and decreased intrinsic function in the right gyrus rectus, right superior/middle frontal gyrus, and left inferior frontal gyrus (Ma et al., 2025). Another rs-fMRI study found that compared with satiated state, abstinent smokers showed altered functional connectivity (FC) between striatal and cingulo-insular network (Sweitzer et al., 2016). However, few studies have focused on comparing the difference of brain function between abstinent smokers and nonsmokers using rs-fMRI, specifically exploring the altered brain activity associated with nicotine withdrawal-induced hyperalgesia. In this study, we used rs-fMRI to compare regional brain activity/connectivity—assessed via fractional amplitude of low-frequency fluctuation (fALFF; 0.01–0.1 Hz), regional homogeneity (ReHo), and rs-FC—between abstinent smokers and nonsmokers. To explore the relationship between altered regional brain activity/connectivity and hyperalgesia, we measured preoperative pain thresholds and the postoperative analgesic requirements in all patients. We hypothesized that nicotine abstinence would lead to specific alterations in regional brain activity/connectivity, which could be associated with lower pain thresholds and increased analgesic use in abstinent smokers. We explored the relationship between smoking behaviors and pain thresholds or postoperative analgesic requirements, as well as the role of different brain regions. These findings may suggest that altered brain activity contributes to nicotine withdrawal-induced hyperalgesia and could offer novel insights into managing postoperative pain in this population.

## Materials and Methods

### Study design

This observational study was conducted at Shanghai Eastern Hepatobiliary Surgery Hospital, China. The study protocol was approved by the Institutional Ethics Committee (Shanghai Eastern Hepatobiliary Surgery Hospital; approval number: EHBHKY2017-03-007) and registered with the Chinese Clinical Trial Registry (ChiCTR-ROC-17011004, https://www.chictr.org.cn) on March 28, 2017. Written informed consent was obtained from all participants before enrolment. All procedures were performed in accordance with the Declaration of Helsinki. Male patients scheduled for partial hepatectomy due to liver masses were assigned to either the abstinent smoker group or nonsmoker group on the day before surgery.

### Participants and study process

Patients were eligible for inclusion if they met the following criteria: (1) male; (2) aged 18–60 years; (3) right-handed; (4) no history of substance abuse other than nicotine; (5) no history of brain lesions or trauma; (6) no communication barriers and capable of completing all assessments; and (7) classified as American Society of Anesthesiologists Grade I or II and Child–Pugh Class A for liver function. Exclusion criteria included (1) contraindications to MRI (e.g., nonremovable metallic implants, claustrophobia); (2) history of peripheral or neurological disorders; (3) chronic pain conditions; (4) long-term use of neuroactive medications (e.g., sedatives, opioids, antidepressants); (5) body mass index >28 or <18 kg/m^2^; (6) inability to complete psychophysiological assessments; (7) contraindications to patient-controlled analgesia (PCA); or (8) inability to complete the 48 h postoperative follow-up, including due to severe postoperative complications. Patients who did not meet the predefined criteria for abstinent smokers or nonsmokers were also excluded.

**Figure 1.**
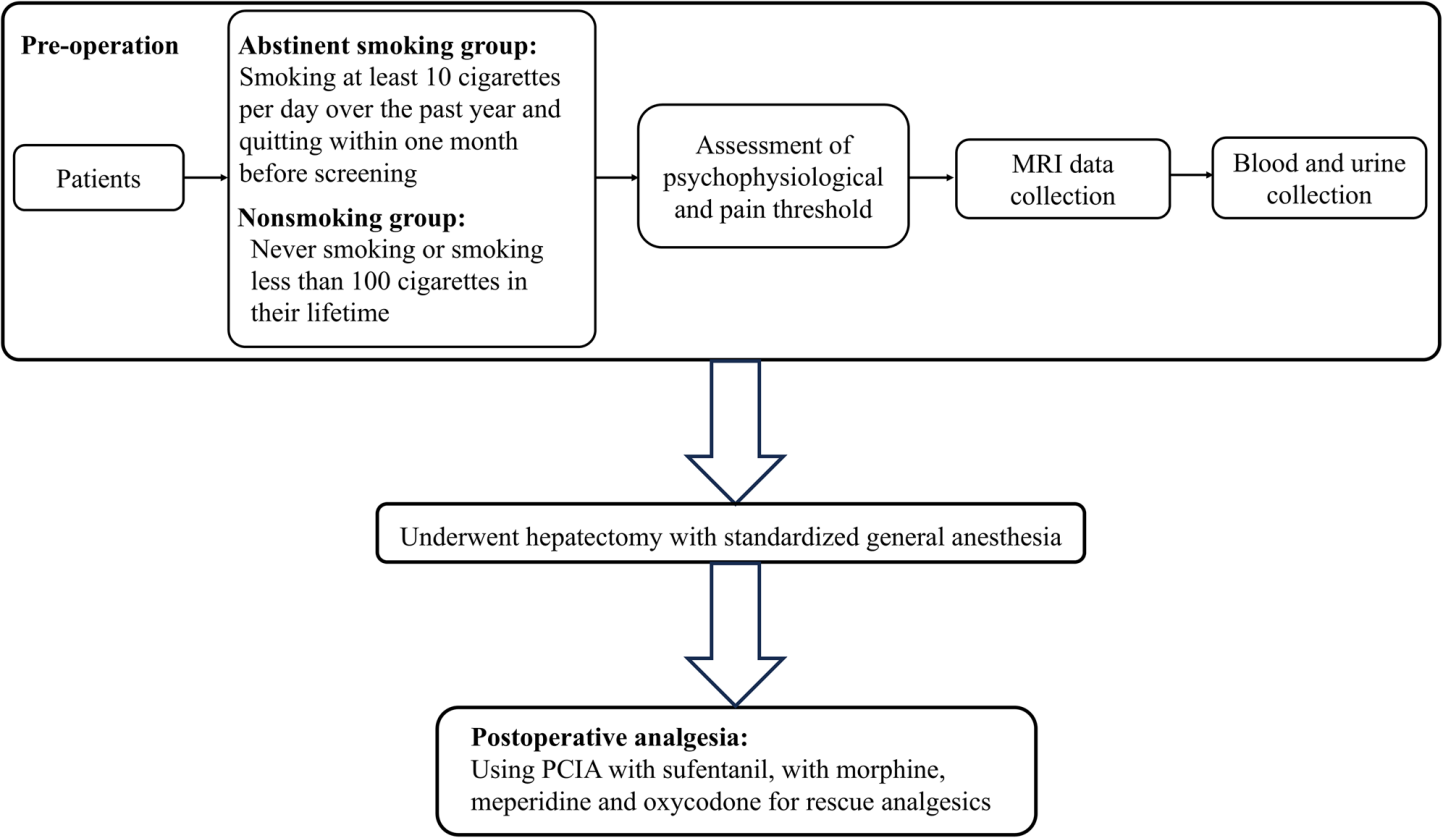
Study flow chart. MRI, magnetic resonance imaging; PCA, patient-controlled analgesia.

The study workflow is illustrated in Figure 1. Patients were assigned to either the nonsmoker or abstinent smoker group based on their smoking status. In brief, nonsmokers were defined as individuals who had never smoked or had smoked <100 cigarettes in their lifetime. Evidence from our previous work showed that smokers who have abstained for <1 month exhibit lower pain thresholds (Shen et al., 2017). Consistently, Hooten et al. reported that current smokers required higher opioid doses at admission to complete a pain rehabilitation program than former smokers who had abstained for at least 1 month and nonsmokers (Hooten et al., 2008). As part of standard preoperative care, all smokers scheduled for elective surgery were asked to quit smoking to minimize postoperative complications. Consequently, abstinent smokers in this study were defined as patients who had regularly smoked at least 10 cigarettes per day over the past year and had quit within 1 month before screening. Individuals abstinent for greater than 1 month were excluded. We also excluded patients who used pharmacological cessation aids—such as nicotine replacement therapy or varenicline—during the perioperative period, because our earlier research suggests that nicotine replacement can alleviate nicotine withdrawal-induced hyperalgesia (C. Zhu et al., 2023).

A team of three independent researchers collected all experimental data 1 d before surgery. The first researcher recorded baseline information and conducted all psychophysiological assessments. The second researcher, blinded to group assignment, performed the pain threshold assessment. The third researcher, also blinded to group assignment, collected the fMRI data with the assistance of technicians. On the following day, all patients underwent hepatectomy under standardized general anesthesia. A standardized postoperative analgesic protocol was applied to all patients to allow for comparison of analgesic consumption. The anesthesiologists responsible for perioperative care were blinded to group assignments.

### Psychophysiological assessments

All patients completed a series of psychophysiological assessments 1 d before surgery, based on their current feelings. Specifically, state and trait anxiety were evaluated using the State–Trait Anxiety Inventory, and depressive symptoms were measured using the Beck Depression Inventory. Pain sensitivity was evaluated with the Pain Sensitivity Questionnaire (PSQ), which reflects an individual's natural disposition to perceive pain. Additionally, abstinent smokers completed three smoking-related questionnaires. The Fagerstrom Test for Nicotine Dependence (FTND) was used to evaluate the severity of nicotine dependence based on preabstinence smoking behavior. Current withdrawal symptoms and nicotine cravings were assessed using the Minnesota Nicotine Withdrawal Scale (MNWS) and the Questionnaire of Smoking Urges (QSU), respectively (Cho et al., 2024).

### Assessment of pain thresholds

Our previous study (Shen et al., 2017) demonstrated that abstinent smokers exhibited lower pain thresholds when assessed using electrical stimulation. Based on this finding, we employed an electrical stimulator to measure the electrical pain threshold (EPT) 1 d before surgery. To further validate the increased pain sensitivity in abstinent smokers, we assessed mechanical pain threshold (MPT) using von Frey filaments on the same day, as this method is convenient, clinically applicable, and well tolerated. Both assessments were noninvasive, time-efficient, and suitable for preoperative evaluation. Specifically, electrical stimuli were delivered using a constant current stimulator (model DS7A; Digitimer) through a pair of electrodes placed on the index finger of the left hand (interelectrode distance, 1 cm; pulse width, 2 ms). The method of limits (in steps of 0.1 mA) was applied to determine the stimulus intensity at which the patient started to feel pain. This procedure was repeated three times, and the mean stimulus intensity was defined as the EPT for each patient. Mechanical stimuli were delivered to the dorsum of the left hand using Semmes–Weinstein monofilaments (Touch-Test 20 Piece Kit; North Coast Medical), with evaluator sizes ranging from 1.65 to 6.65 and target forces from 0.008 to 300 g (Keizer et al., 2007). The filament was pressed against the skin at a 90° angle until it bowed and then held in place for 1.5 s. The method of limits was used to determine the evaluator size at which pain was first reported (increasing sequence) and subsequently lost (decreasing sequence). This process was repeated three times, and the geometric mean of the evaluator size values was defined as the MPT for each patient.

### Confirmation of smoking status

We measured the cotinine levels in both plasma and urine to verify the presumed difference between the two groups using previously reported methods (Shen et al., 2017). Specifically, 1 d before surgery, blood and urine specimens were obtained, labeled, centrifuged at room temperature, and promptly stored at −80°C. After study completion, all samples were transferred to the pharmacy department for analysis. Cotinine levels in the serum and urine were determined using ultrapressure liquid chromatography–tandem mass spectrometry (1290 Infinity II series LC system, G6470 triple quadrupole mass spectrometer; Agilent Technologies), following an optimized protocol established in a previous study (Wei et al., 2014). The limit of quantification of the method was 0.1 ng/ml. The accuracy and precision of the method were within 8% of the relevant concentration range.

### Anesthesia process and postoperative analgesia

All patients underwent hepatectomy with standardized general anesthesia, as reported previously (Shen et al., 2017; Wei et al., 2023). In brief, tracheal intubation was implemented after anesthesia induction with intravenous midazolam (0.03 mg/kg), target-controlled infusion of propofol (target plasma concentration of 5.0 µg/ml achieved using the Paedfusor model), sufentanil (0.3 µg/kg), and *cis*-atracurium (0.2 mg/kg). Anesthesia was maintained with propofol (target plasma concentration of 3.0–5.0 µg/ml), remifentanil (0.1–0.2 µg/kg/min), and *cis*-atracurium (1.5 µg/kg/min) to maintain the bispectral index in the range of 40–60 and the heart rate and blood pressure to within 20% of preoperative values. Mechanical ventilation was adjusted to maintain the end-tidal partial pressure of carbon dioxide to between 35 and 45 mm Hg, with a tidal volume of 8–10 ml/kg and a respiratory rate of 10–14 breaths/min.

Postoperative analgesia was administered in a manner consistent with our previous report (Shen et al., 2017). Following anesthesia, patients were transferred to a postanesthesia care unit for ongoing care. All patients received sufentanil (1 µg/ml; Humanwell Pharmaceutical) via a PCA device. The PCA device was programmed to deliver 2 ml of analgesics as an intravenous bolus, with a lockout time of 10 min and a background infusion rate of 2 ml/h. The maximum allowable sufentanil dosage was capped at 10 µg/h. If patients exhibited poor response to sufentanil, supplemental rescue analgesics were used to relieve postoperative pain. The analgesics administered in this study were injectable morphine, meperidine, and oxycodone. The doses of these opiate analgesics were converted into morphine-equivalent doses based on equianalgesic dose ratios.

### Data collection

The fMRI data were collected using a 3.0 Telsa MRI system (Discovery MR 750; General Electric Healthcare) with an eight-channel receive–only radiofrequency head coil array 1 d before surgery. A standard birdcage head coil, along with restraining foam pads, was used to minimize head motion and scanner noise. A T1-weighted structural image was acquired using a three-dimensional spoiled gradient recalled echo sequence with the following parameters: repetition time, 6.896 ms; echo time, 2.99 ms; flip angle, 8°; in-plane matrix size, 256 × 256; 176 slices; field of view, 256 × 256 mm^2^; in-plane resolution, 1 × 1 mm^2^; and slice thickness, 1 mm. The rs-fMRI images were acquired using an echoplanar imaging sequence using the following parameters: repetition time, 2,000 ms; echo time, 30 ms; flip angle, 90°; field of view, 64 × 64 mm^2^; data matrix, 64 × 64; in-plane resolution, 3 × 3 mm^2^; slice thickness, 3.5 mm; slice spacing, 0.5 mm; and 256 volumes. The rs-fMRI scan took ∼10 min. During the rs-fMRI data acquisition, patients were instructed to keep their eyes closed, remain still, and not think about anything systematically.

Patient characteristics, including age, height, weight, and smoking-related behaviors, were obtained via standardized questionnaires. Medical information such as blood test results, anesthesia records, and surgical data was retrieved from electronic medical records. Postoperative analgesic requirements at 1, 6, 24, and 48 h after surgery were recorded by a trained research assistant who was blinded to group assignment. To account for variations in the body size, all analgesic doses were normalized by dividing the total dose by the patient's body weight.

### Fs-fMRI data preprocessing

The rs-fMRI data were analyzed using Data Processing and Analysis for (Resting-State) Brain Imaging (DPABI; version V8.2_240510, https://rfmri.org/DPABI) toolbox (Yan et al., 2016), implemented in MATLAB 2023b (MathWorks). The first 10 volumes were discarded to minimize the effects of spin saturation and participant adaptation to the scanning environment. The remaining 246 volumes were slice-time corrected and realigned for head motion using a six-parameter rigid body transformation. Patients with head movements exceeding 2.0 mm in translation or 2.0° in rotation were excluded. Individual structural images were coregistered to the mean functional image and segmented into gray matter, white matter, and cerebrospinal fluid. Nuisance covariates—including white matter signal, cerebrospinal fluid signal, and head motion scrubbing parameters (Friston 24)—were regressed out from the fMRI data. Please note that, time points with a framewise displacement (Jenkinson FD) exceeding 0.2 were classified as “bad.” The scrubbing regressor was defined to include the identified bad time points, along with one preceding and two subsequent time points. The processed fMRI data were then normalized to standard space using DARTEL (resampling voxel size, 3 × 3 × 3 mm^3^) and linearly detrended to remove drift. The resulting data were used for subsequent analyses.

### FALFF, ReHo, and rs-FC analyses

The DPABI toolbox was used to compute fALFF, ReHo, and rs-FC from the preprocessed rs-fMRI data. For fALFF calculation, data were spatially smoothed using a 4 mm full-width at half-maximum (FWHM) Gaussian kernel. The relative amplitude of low-frequency fluctuations within the 0.01–0.1 Hz range was then calculated. Standardized fALFF values were obtained by normalizing each voxel's fALFF to the global mean and were used for subsequent statistical analysis. For ReHo, Kendall's coefficient of concordance (KCC) was used to measure the local synchronization of the time series between each voxel and its 26 neighboring voxels, after bandpass filtering (0.01–0.1 Hz). The resulting ReHo values were standardized by dividing each voxel's KCC by the mean KCC of the whole brain and then spatially smoothed with a 4 mm FWHM Gaussian kernel.

Brain regions showing significant group differences in fALFF and ReHo were defined as regions of interest (ROIs) for ROI-based rs-FC analysis. The preprocessed data were smoothed with a 4 mm FWHM Gaussian kernel and bandpass filtered (0.01–0.1 Hz). Pearson's correlation coefficients were calculated between the mean time series of each ROI and those of all other brain voxels. These correlation maps were then transformed to Fisher's *Z* scores (zFC maps) for further statistical analyses.

### Statistical analysis

The statistical analysis plan was determined before participant enrolment. Descriptive statistics are presented as means (standard deviations), medians (interquartile ranges), or frequencies (proportions), depending on the data type and distribution. For continuous variables, normality was confirmed using the Kolmogorov–Smirnov test. Group comparisons of continuous variables were conducted using Student's *t* test or the Mann–Whitney *U* test, as appropriate. Repeated measures were compared using repeated-measure analysis of variance (ANOVA), with post hoc tests performed when a significant interaction effect was detected. Categorical variables were compared using the *χ*^2^ test. To explore the associations between smoking behaviors (i.e., cigarettes/day, smoking duration, abstinence duration, FTND, MNWS, QSU, and cotinine levels) and pain thresholds or postoperative analgesic requirements, we performed Pearson's or Spearman’s correlation analyses in abstinent smokers, selected based on data distribution. The level of statistical significance was set at *p* < 0.05. All statistical analyses were performed using SPSS 22.0 (SPSS).

To examine group differences in fALFF, ReHo, and FC, two-sample *t* tests were performed using DPABI in MATLAB 2023b, with age and head motion (mean framewise displacement) included as covariates. Correlation analyses were used to explore associations between whole-brain regional fMRI maps and pain thresholds or postoperative analgesic requirements in both abstinent smokers and nonsmokers. Multiple comparisons were conducted using Gaussian random field correction [voxel-level, *p* < 0.001; cluster-level, *p* < 0.05 (two-tailed)] for the fALFF, ReHo, rs-FC, and correlation analyses. Furthermore, mediation analyses tested whether pain-associated brain regions mediated the effects of smoking behaviors on pain thresholds and postoperative analgesic requirements.

## Results

### Study population

Sixty right-handed male patients scheduled for surgery due to liver masses were enrolled in the study between April 1, 2017, and November 8, 2017. Among them, 30 were nonsmokers (nonsmoking group), and 30 were smokers who had been asked to quit smoking upon hospitalization (abstinent smoker group). There were no significant differences in demographic and clinical characteristics between the groups, except for white blood cell count (Table 1). Plasma and urine cotinine concentrations were significantly higher in the abstinent smoker group compared with the nonsmoker group (Table 1), confirming the validity of group classification based on smoking history. Details on smoking behavior, nicotine dependence severity, and withdrawal symptoms in the abstinent smoker group are shown in Table 2.

**Table 1.** Patient characteristics of the abstinent smoker and nonsmoker groups

Variables	Abstinent smoking group	Nonsmoking group	*p* value
	(*n* = 30)	(*n* = 30)	
Basic clinical characteristics			
Age, years	52.60 (9.62)	51.60 (8.46)	0.67
Body mass index, kg/m^2^	23.18 (2.77)	24.22 (2.12)	0.11
ASA I/II	17/13	18/12	0.79
Systolic blood pressure, mmHg	121.63 (11.16)	126.20 (13.65)	0.16
Diastolic blood pressure, mmHg	75.17 (10.01)	77.50 (11.39)	0.40
Anesthesia and surgery related			
Operation time, min	155.67 (28.61)	148.33 (28.65)	0.33
Intraoperative fluid, ml	1,966.67 (376.31)	2,010.00 (336.67)	0.64
Intraoperative blood loss, ml	281.67 (123.51)	235.00 (108.40)	0.13
Intraoperative urine, ml	257.00 (105.02)	273.33 (87.82)	0.52
Blood test indicators			
Red blood cell, ×10^12^/L	4.75 (0.45)	4.93 (0.53)	0.18
White blood cell, ×10^12^/L	5.88 (1.74)	4.77 (1.12)	0.004*
Platelet, ×10^9^/L	156.67 (57.63)	160.89 (59.74)	0.83
Hemoglobin, g/L	146.90 (14.02)	147.93 (13.45)	0.77
Total bilirubin, μmol/L	13.08 (5.72)	15.04 (7.12)	0.24
Direct bilirubin, μmol/L	6.39 (3.81)	6.72 (3.55)	0.72
Albumin, g/L	40.87 (3.77)	42.55 (3.82)	0.09
Alanine transaminase, U/L	38.60 (29.67)	28.30 (19.32)	0.12
Glutamic oxaloacetylase, U/L	33.93 (22.01)	29.93 (25.89)	0.52
Creatinine, μmol/L	79.93 (12.49)	80.23 (12.12)	0.93
Glomerular filtration rate, ml/min	96.61 (17.25)	94.75 (26.67)	0.75

ASA, American Society of Anesthesiologists. Data are presented as means (standard deviations) or numbers. Group differences for continuous variables were evaluated using unpaired *t* tests or the Mann–Whitney *U* test. Group differences for categorical variables were tested using the chi-square test. *p* < 0.05 was considered statistically significant.

* *p* < 0.05.

**Table 2.** Psychological variables, pain sensitivity, and smoking behaviors in the abstinent smoker and nonsmoker groups

Variables	Abstinent smoking group	Nonsmoking group	Statistical variables	*p* value
	(*n* = 30)	(*n* = 30)	*t*/*F*/*U* value	
Psychological variables				
SAI	37.90 (13.11)	32.97 (11.65)	−1.54	0.13
TAI	38.93 (11.13)	33.13 (11.70)	−1.97	0.05
BDI	4.50 (4.89)	2.47 (2.69)	−2.00	0.05
Pain sensitivity				
PSQ	67.30 (30.86)	44.90 (21.21)	−3.28	0.002**
Pain threshold, mA^a^	1.28 (0.45)	1.76 (1.06)	2.30	0.03*
Pain threshold, g^b^	3.67 (3.24)	83.46 (140.92)	184^d^	<0.001***
Smoking behaviors				
Cigarettes per day, numbers	21.83 (11.78)	-		-
Duration of smoking, years	28.87 (10.42)	-		-
Duration of abstinence, days	5.37 (4.18)	-		-
FTND	5.23 (2.49)	-		-
MNWS score	5.21 (5.53)	-		-
QSU-Brief	28.43 (14.47)			
Cotinine in urine, ng/ml	142.00 (192.90)	0.85 (1.40)	872^d^	<0.001***
Cotinine in plasma, ng/ml	10.96 (9.53)	1.52 (2.74)	798.5^d^	<0.001**
Postoperative analgesic requirements, mg/kg^c^			9.37^e^	<0.001***
1 h	0.22 (0.10)	0.16 (0.09)	0.82	>0.99
6 h	0.56 (0.20)	0.42 (0.12)	2.28	0.09
24 h	1.54 (0.38)	1.19 (0.25)	5.44	<0.001***
48 h	2.31 (0.36)	2.08 (0.28)	3.67	0.001**

SAI, State Anxiety Inventory; TAI, Trait Anxiety Inventory; BDI, Beck Depression Inventory; PSQ, Pain Sensitivity Questionnaire; FTND, Fagerstrom Test for Nicotine Dependence; MNWS, Minnesota Nicotine Withdrawal Scale; QSU, Questionnaire of Smoking Urges. Data are presented as means (standard deviations). Group differences for continuous variables were evaluated with unpaired *t* tests or the Mann–Whitney *U* test. Pain thresholds were evaluated using an electrical stimulator^a^ and von Frey filaments^b^.

c Repeated analysis of variance (repeated ANOVA) was used for analysis, and post hoc comparisons were performed with post hoc Bonferroni’s test to compare group differences at each time point.

d Represents *U* values calculated from Mann–Whitney *U* test.

e Represents *F* values of interaction effect calculated from repeated ANOVA.

* *p* < 0.05.

** *p* < 0.01.

*** *p* < 0.001.

### Lower pain threshold and more postoperative analgesic requirements in abstinent smokers

**Figure 2.**
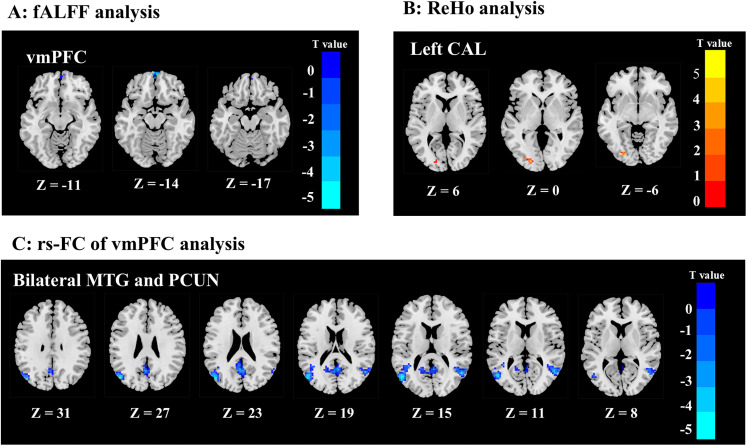
Brain regions with altered regional activity and FC in abstinent smokers. ***A***, The voxel-wise fALFF results suggested that bilateral medial vmPFC activity was significantly lower in the abstinent smoker group than in the nonsmoker group. ***B***, The voxel-wise ReHo results suggested that left CAL activity was significantly higher in the abstinent smoker group than in the nonsmoker group. ***C***, The ROI-based rs-FC analysis revealed that rs-FC of vmPFC to bilateral MTG and PCUN was lower in abstinent smokers.

As shown in Table 2, compared with nonsmokers, abstinent smokers exhibited higher PSQ scores (*t* = −3.28; *p* = 0.002; 95% CI, 8.72–36.08), lower EPT (*t* = 2.30; *p* = 0.03; 95% CI, −0.90 to −0.06), and lower MPT (*U* = 184; *p* < 0.001; 95% CI, −27.19 to −1.38), indicating higher pain sensitivity and lower pain threshold in abstinent smokers before surgery. In addition, repeated-measure ANOVA revealed a significant group × time interaction for postoperative analgesic requirements (*F*_[3,174]_ = 9.37; *p* < 0.001; Fig. 2*D*). Post hoc tests indicated that abstinent smokers required significantly more analgesics than nonsmokers at both 24 h (95% CI, 0.19–0.51; *p* < 0.001, Bonferroni’s corrected) and 48 h (95% CI, 0.07–0.40; *p* = 0.0012, Bonferroni’s corrected) postoperatively. These results suggest that, compared with nonsmokers, abstinent smokers had lower pain thresholds and greater postoperative analgesic requirements.

### Altered regional activity and FC in abstinent smokers

After applying exclusion criteria related to head movement, 58 participants (29 abstinent smokers and 29 nonsmokers) remained eligible for rs-fMRI analysis. Group differences in fALFF and ReHo are illustrated in Figure 2 and summarized in Table 3. Compared with nonsmokers, abstinent smokers showed significantly lower fALFF in the bilateral ventromedial prefrontal cortex (vmPFC; Fig. 2*A*), with a total cluster size of 13 voxels. In contrast, ReHo was significantly higher in the left calcarine (CAL; Fig. 2*B*), with a cluster of 34 voxels, in abstinent smokers relative to nonsmokers. These two brain regions were subsequently defined as ROIs for further rs-FC analysis. Compared with nonsmokers, abstinent smokers exhibited significantly lower rs-FC between vmPFC and both the bilateral middle temporal gyrus (MTG) and the precuneus (PCUN; Fig. 2*C*,*D*; Table 4). No other group differences reached statistical significance. These findings suggest that abstinent smokers and nonsmokers differ in preoperative regional brain activity and FC patterns.

**Table 3.** Brain regions with significant differences in fMRI measures (fALFF, ReHo, and rs-FC) between abstinent smokers and nonsmokers

Anatomical regions	Regions in AAL_3 templates	Peak MNI coordinates			*T*_max_	Voxels
		*x*	*y*	*z*		
fALFF						
Bilateral vmPFC	Frontal_Med_Orb_L/R	3	63	−15	−4.7096	13
ReHo						
Left CAL cortex	Occipatal_Mid_L	−21	−87	0	4.7454	34
rs-FC to vmPFC						
Bilateral MTG	Temporal_Mid_R	51	−60	15	−5.8299	97
	Temporal_Mid_L	−51	−66	15	−5.9066	181
Bilateral PCUN	Precuneus_L/R	−3	−60	24	−5.087	201

fALFF, fractional amplitude of low-frequency fluctuation; ReHo, regional homogeneity; rs-FC, resting-state functional connectivity; MNI, Montreal Neurological Institute. Results are Gaussian random field-corrected [voxel-level, *p* < 0.001; cluster-level *p* < 0.05 (two-tailed)].

**Table 4.** Correlation between regional fMRI activity and pain thresholds and postoperative analgesic requirements at 48 h postsurgery in the abstinent smokers

Anatomical regions	Regions in AAL_3 templates	Peak MNI coordinates			*T*_max_	*R* value	Voxels
		*x*	*y*	*z*			
Correlations between fMRI and pain threshold							
fALFF and EPT							
Right CAL cortex (CAL)	Calcarine_R	18	−72	12	4.4596	0.665	20
ReHo and EPT							
Left CAL cortex (CAL)	Calcarine_L	−12	−75	21	4.668	0.682	43
Left PCC	Cingulum_Mid_L	−15	−33	42	5.970	0.766	36
rs-FC and MPT							
Right DLPFC	Frontal_Mid_R	42	42	9	6.356	0.785	32
	Frontal_Inf_Tri_R/Frontal_Inf_Oper_R	51	30	27	5.903	0.763	95
Correlations between fMRI and postoperative analgesic requirements at 48 h							
fALFF							
Left vmPFC	Frontal_Med_Orb_L/Cingulum_Ant_L	−9	42	0	−5.361	−0.730	40
	Frontal_Med_Orb_R	15	45	−12	−5.076	−0.710	23
rs-FC							
Bilateral medial and middle orbital frontal cortex (OFC)	Frontal_Sup_Orb_L/Frontal_Mid_Orb_L	−15	42	−12	4.893	0.699	112
	Frontal_Sup_Orb_R	12	33	−6	5.001	0.707	83

fMRI, functional magnetic resonance imaging, fALFF, fractional amplitude of low-frequency fluctuation; ReHo, regional homogeneity; rs-FC, resting-state functional connectivity; MNI, Montreal Neurological Institute. Results are Gaussian random field-corrected [voxel-level, *p* < 0.001; cluster-level, *p* < 0.05 (two-tailed)].

### Association among smoking behaviors, brain function, and pain thresholds

**Figure 3.**
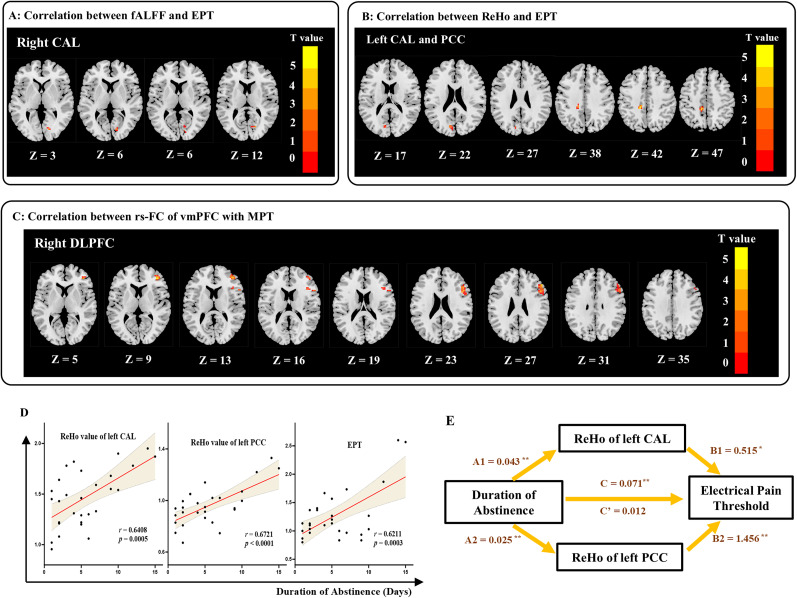
Association between pain thresholds and whole-brain rs-fMRI. ***A***, EPT was positively correlated with fALFF value in the right CAL cortex (CAL). ***B***, EPT were positively correlated with the ReHo value in the left CAL and the left PCC. ***C***, MPT was positively correlated with rs-FC of the bilateral vmPFC to the right DLPFC. ***D***, The scatterplots showed the correlation between duration of abstinence and ReHo values in the left CAL and left PCC and between duration of abstinence and EPT. The shaded regions represent 95% credibility interval. ***E***, The mediation effect of left CAL and PCC in association with duration of abstinence and EPT. A1, A2, effect of duration of abstinence on mediator. B1, B2, effect of mediator on EPT. C, total effect of duration of abstinence on EPT. C’, direct effect of duration of abstinence on EPT.

Whole-brain correlation analysis between functional fMRI maps with pain thresholds (Table 4) showed that in the abstinent smoker group, EPT was significantly positively correlated with fALFF values (Fig. 3*A*) in the right CAL, with a cluster size of 20 voxels. For ReHo (Fig. 3*B*), significant positive correlations were found between EPT and ReHo in the left CAL (cluster size of 43 voxels) and the left posterior cingulate cortex (PCC; cluster size of 36 voxels). No significant correlations were found between EPT and rs-FC values. However, as shown in Figure 3*C*, MPT was significantly positively correlated with rs-FC from the vmPFC to the right dorsolateral prefrontal cortex (DLPFC). No significant correlations were found between MPT and either fALFF or ReHo values. In the nonsmoking group, no significant correlations were observed between brain activity/FC and either EPT or MPT.

Next, we explored the correlations between smoking behaviors (i.e., cigarettes/day, smoking duration, abstinence duration, FTND, MNWS, QSU, and plasma/urine cotinine levels) and the brain regions associated with pain thresholds. Duration of abstinence was positively correlated with ReHo values in both the left CAL and left PCC (Fig. 3*D*). Notably, duration of abstinence was also significantly positively correlated with EPT (*r* = 0.6211; *p* = 0.0003; Fig. 3*D*). Based on these findings, we hypothesized that the lower pain thresholds observed in abstinent smokers may be mediated by altered regional activity in the left CAL and left PCC. To test this hypothesis, a mediation analysis was conducted, with duration of abstinence as independent variable, EPT as dependent variable, ReHo values in the left CAL and left PCC as mediators, and age as a covariate. The results indicated that both the left CAL and left PCC fully mediated the relationship between duration of abstinence and EPT in abstinent smokers (Fig. 3*E*).

### Association among smoking behaviors, brain function, and postoperative analgesic requirements

**Figure 4.**
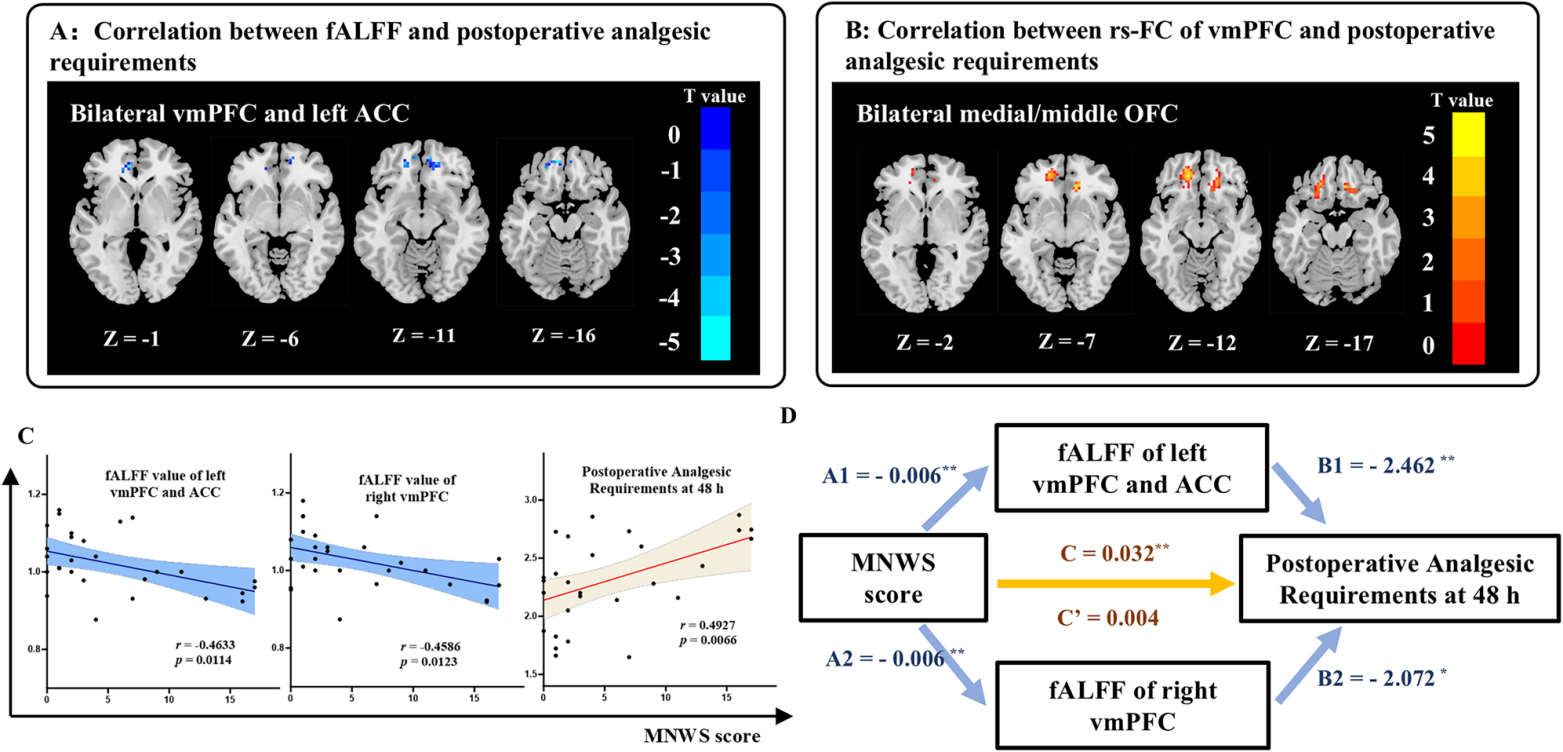
Association between postoperative analgesic requirements and whole-brain rs-fMRI. ***A***, Postoperative analgesic requirements at 48 h was negatively correlated with fALFF values in the left ACC and bilateral vmPFC. ***B***, Postoperative analgesic requirements at 48 h was positively correlated with rs-FC of vmPFC to the bilateral medial and middle orbital frontal cortex (OFC). ***C***, The scatterplots showed the correlation between MNWS with fALFF value in the right vmPFC, and left vmPFC and ACC, and between MNWS and postoperative analgesic requirements at 48 h. The shaded regions represent 95% credibility interval. ***D***, The mediation effect of vmPFC and ACC in association with MNWS score and postoperative analgesic requirements at 48 h. A1, A2, effect of duration of abstinence on mediator. B1, B2, effect of mediator on EPT. C, total effect of duration of abstinence on EPT. C’, direct effect of duration of abstinence on EPT.

The result of whole-brain correlation analysis between postoperative analgesic requirements at 48 h and functional fMRI maps are shown in Figure 4, *A* and *B*, and Table 4. In the abstinent smoker group, significant negative correlations were found between postoperative analgesic requirements and fALFF values in the left anterior cingulate cortex (ACC) and bilateral vmPFC (Fig. 4*A*). Additionally, a significant positive correlation was found between postoperative analgesic requirements and rs-FC between the vmPFC with the bilateral medial/middle orbitofrontal cortex (OFC; Fig. 4*B*; Table 4). No significant correlations were detected for ReHo values. Similarly, no significant correlations were found between postoperative analgesic requirements and regional fMRI activity in the nonsmoking group.

To further explore the relationship between smoking behavior and the brain regions associated with postoperative analgesic requirements in abstinent smokers, we conducted a correlation analysis. The results showed that the MNWS score was negatively correlated with the fALFF value in left ACC and bilateral vmPFC (Fig. 4*C*). Moreover, the MNWS score was positively correlated with postoperative analgesic requirements (Fig. 4*C*). Based on these findings, we hypothesized that increased postoperative analgesic requirements in abstinent smokers may be mediated by altered regional activity in the left ACC and bilateral vmPFC. To test this hypothesis, we conducted a mediation analysis, with the MNWS score as independent variable, postoperative analgesic requirements at 48 h as dependent variable, fALFF values in the left ACC and bilateral vmPFC as mediators, and age as a covariate. The analysis revealed that the left ACC and bilateral vmPFC fully mediated the relationship between MNWS scores and postoperative analgesic requirements in abstinent smokers (Fig. 4*D*).

## Discussion

In this study, abstinent smokers exhibited lower pain thresholds, increased postoperative analgesic requirements, and altered neural activity—specifically lower fALFF in the vmPFC, higher ReHo in the left CAL, and decreased FC between vmPFC and bilateral MTG/PCUN. These findings are consistent with previous reports (Garrison et al., 2017; Kim et al., 2017; Shen et al., 2017; Ditre et al., 2018; Wang et al., 2018; Zhao et al., 2018; Wei et al., 2023; Shepherd et al., 2024). Furthermore, abstinent smokers—unlike nonsmokers—showed a positive correlation between EPT and fALFF in the right CAL and between EPT and ReHo in the left CAL and PCC. Rs-FC of vmPFC was also positively correlated with MPT in right DLPFC. Pain thresholds (EPT) correlated positively with abstinence duration, an association fully mediated by CAL and PCC activity. Meanwhile, abstinent smokers showed a negative correlation between postoperative analgesic requirements and fALFF in vmPFC and left ACC and positively with rs-FC from vmPFC to bilateral medial/middle OFC. Withdrawal symptoms positively predicted postoperative analgesic requirements and mediated by vmPFC and ACC activity. While prior studies have described structural or functional neural changes in nicotine dependence and addiction (Sutherland et al., 2016; Garrison et al., 2017; Sutherland and Stein, 2018; Wang et al., 2018; Zhao et al., 2018; Zhang and Volkow, 2019), this is the first study to link fMRI-based neural alterations to hyperalgesia in abstinent smokers accessed by both pain thresholds and postoperative analgesic requirements.

The default mode network (DMN)—a key resting-state network implicated in pain processing—typically encompasses three components: a midline core network, a medial temporal subsystem (MTL-DMN), and a dorsal medial prefrontal subsystem (dMPFC-DMN). The midline core network consists of the medial prefrontal cortex (mPFC) and PCC/PCUN (Zhang and Volkow, 2019). We observed reduced activity and rs-FC in midline core network of DMN in abstinent smokers. Mediation analyses indicated that PCC and vmPFC activity contributed to elevated pain sensitivity and increased postoperative analgesic requirements, aligning with reports of DMN abnormalities in pain modulation (Kucyi et al., 2014; Hsiao et al., 2017), nicotine addiction, withdrawal, and relapse (Menossi et al., 2013; Sutherland et al., 2016; Zhang and Volkow, 2019; Yip et al., 2022; Zhang et al., 2024). The current study underscored the role of DMN dysfunction in nicotine withdrawal-induced hyperalgesia and suggested its potential as a therapeutic target for managing postoperative pain in abstinent smokers. Interestingly, different DMN components mediated heightened pain sensitivity before surgery and increased postoperative analgesic requirements, indicating distinct mechanisms. Indeed, the pain threshold measured in our study reflects acute, transient pain—elicited by the activation of nociceptive transducers without tissue injury—whereas postoperative pain results from substantial tissue damage (Loeser and Melzack, 1999; Yarnitsky et al., 2013).

VmPFC, as the anterior midline core network in DMN, plays an important role in both nicotine withdrawal symptoms (Cole et al., 2020; Huang et al., 2023) and pain modulation (Ong et al., 2019; Pan et al., 2023), respectively. Short-term abstinence affects prefrontal activity related to cue reactivity (Goldstein and Volkow, 2011), and neuromodulation of the DLPFC and vmPFC can alleviate withdrawal-related cognitive deficits (Fischell et al., 2020). Agonism of nicotinic acetylcholine receptors improves cognition by modulating mPFC activity (Sutherland et al., 2015). Animal studies further support that nicotine withdrawal alters mPFC structure and function, leading to withdrawal symptoms such as impaired cognitive memory, anxiety, and hyperalgesia (Huang et al., 2022, 2023; Shao et al., 2023; Gozen et al., 2024). The mPFC also regulates various types of pain (Cheriyan and Sheets, 2018; Ong et al., 2019; Fang et al., 2023; Mosch et al., 2023; Lv et al., 2024), and its activity may even predict spontaneous pain (Shirvalkar et al., 2023).

We also found reduced rs-FC between vmPFC and PCUN—a midline core network in DMN—in abstinent smokers. Notably, analysis of data from the “1000 Functional Connectomes” project revealed that the strongest FC hubs in the brain are concentrated in the midline core regions of the DMN, specifically encompassing the mPFC and PCC/PCUN (Zhang and Volkow, 2019). The decrease of FC strength between mPFC and PCC/PCUN were associated with some of diseases such as schizophrenia (Zong et al., 2019). Altered PCUN activity and connectivity are reported in chronic pain conditions (Yu et al., 2023; Chen et al., 2025; Lai et al., 2025; Wu et al., 2025), and PCC/PCUN—an important hub in DMN (Fransson and Marrelec, 2008)—is linked to neuropathic pain (J. Zhu et al., 2023) and headache (Biggs et al., 2020; Ou et al., 2024). Our results found a decreased rs-FC between vmPFC and PCUN, and PCC mediated the association between duration of abstinence and pain thresholds in abstinent smokers, inferring that nicotine deprivation disrupts vmPFC—PCC/PCUN connectivity, contributing to hyperalgesia.

Beyond the DMN core, CAL and ACC also mediated the relationships between abstinence and pain measures. Traditionally, the CAL has been regarded as a principal structure within the primary visual cortex, primarily implicated in visual processing. However, according to the recent research, emerging evidence suggests that several components of the visual cortex, especially CAL, showed functional alterations or structural changes in various pain disorders, including endometriosis-related chronic pelvic pain (Maulitz et al., 2024), primary dysmenorrhea (Jin et al., 2024), brachial plexus avulsion-induced neuropathic pain (J. Zhu et al., 2023), new persistent daily headaches (Qiu et al., 2023), acute pain after total knee arthroplasty (Kang et al., 2023), and chronic migraine (Yuan et al., 2022). Furthermore, aberrant spontaneous activity in the CAL might underlie the mechanism of acupuncture analgesia in chronic stable angina pectoris (Lan et al., 2022). Beyond somatic pain, patients suffering from chronic visceral pain (e.g., irritable bowel syndrome) showed higher spontaneous activity in CAL at resting-state, as well as heightened activation during pain anticipation (Chen et al., 2021). As the discussion in Lan et al.'s paper (Lan et al., 2022), these findings imply that the CAL may be involved not only in pain perception and pain affective processing but also in hypervigilance to pain anticipation. Another recent interesting finding was that the FC from CAL to the right nucleus accumbens (one of reward-related brain regions) was increased after repetitive transcranial magnetic stimulation in nicotine-dependent participants, indicating CAL may be involved in nicotine dependence and withdrawal (Wang et al., 2024). ACC is similarly implicated in nicotine addiction (Sutherland and Stein, 2018; Keeley et al., 2020) and withdrawal symptoms (Fischell et al., 2020), especially hyperalgesia (Long et al., 2023; Chen et al., 2024). Short-term nicotine deprivation alters dorsal ACC glutamate concentration and concomitant cingulate-cortical FC (Abulseoud et al., 2020). These studies, combined with our research, suggest that CAL and ACC may play a certain role in nicotine withdrawal-induced hyperalgesia. More definitive evidence and its neural molecular mechanisms should be further studied in future research.

Notably, electrical (EPT) and mechanical (MPT) pain thresholds correlated with distinct neural markers: EPT with CAL and PCC activity and MPT with vmPFC–DLPFC connectivity. In modern physiology, three subtypes of sensory nerve fibers including Aβ, Aδ, and C fibers have been identified (Lin et al., 2020). The low-intensity electrical stimuli primarily activate Aβ afferent fibers (Yague et al., 2010), while the mechanical stimuli primarily activate Aδ and C fibers (Lee et al., 2011). No such correlations occurred in nonsmokers, suggesting abstinence-specific neural dysfunction in CAL, ACC, and fronto-cingulate pathways underlying hyperalgesia.

Several limitations should be noted. First, only male patients were included. Nicotine withdrawal symptoms vary between the sexes (Sussman et al., 1998; Allen et al., 2018; Wen et al., 2022), with women generally reporting greater pain sensitivity than men (Bartley and Fillingim, 2013). Further studies should include females. Second, this study focused solely on fALFF, ReHo, and rs-FC. Future studies should investigate other neural dynamics, particularly those underlying connectivity among canonical brain networks. Third, as an observational study, mediation analyses suggest but cannot confirm causality—fMRI changes may mediate pain behaviors, but reverse or third-factor causation remains possible. Intervention studies are needed to establish causal links. Finally, given the limited sample size of the current study, it would be valuable to conduct future research with a larger cohort to validate the results.

## Conclusion

This study aimed at exploring the underlying mechanisms of nicotine withdrawal-induced hyperalgesia using fMRI in surgical patients. Clinical analysis showed that abstinent smokers exhibited lower pain thresholds and higher postoperative analgesic requirements than nonsmokers. These differences may be attributed to functional abnormalities of specific brain regions, including vmPFC, CAL, PCC, ACC, as well as rs-FC from vmPFC to MTG, PCUN, DLPFC, and medial/middle OFC. Further studies exploring the neurobiological mechanisms underlying the effects of nicotine abstinence in these brain regions are warranted.

## References

[B1] Abulseoud OA, et al. (2020) Short-term nicotine deprivation alters dorsal anterior cingulate glutamate concentration and concomitant cingulate-cortical functional connectivity. Neuropsychopharmacology 45:1920–1930. 10.1038/s41386-020-0741-9 32559759 PMC7608204

[B2] Allen AM, Abdelwahab NM, Carlson S, Bosch TA, Eberly LE, Okuyemi K (2018) Effect of brief exercise on urges to smoke in men and women smokers. Addict Behav 77:34–37. 10.1016/j.addbeh.2017.09.009 28950116 PMC8691420

[B3] Aspera-Werz RH, Mück J, Linnemann C, Herbst M, Ihle C, Histing T, Nussler AK, Ehnert S (2022) Nicotine and cotinine induce neutrophil extracellular trap formation-potential risk for impaired wound healing in smokers. Antioxidants 11:2424. 10.3390/antiox11122424 36552632 PMC9774423

[B4] Bartley EJ, Fillingim RB (2013) Sex differences in pain: a brief review of clinical and experimental findings. Br J Anaesth 111:52–58. 10.1093/bja/aet127 23794645 PMC3690315

[B5] Beaver JD, Long CJ, Cole DM, Durcan MJ, Bannon LC, Mishra RG, Matthews PM (2011) The effects of nicotine replacement on cognitive brain activity during smoking withdrawal studied with simultaneous fMRI/EEG. Neuropsychopharmacology 36:1792–1800. 10.1038/npp.2011.53 21544072 PMC3154097

[B6] Biggs EE, Timmers I, Meulders A, Vlaeyen JWS, Goebel R, Kaas AL (2020) The neural correlates of pain-related fear: a meta-analysis comparing fear conditioning studies using painful and non-painful stimuli. Neurosci Biobehav Rev 119:52–65. 10.1016/j.neubiorev.2020.09.016 33011229

[B7] Chen D, Shen L, Zhang Y-Z, Kan B-F, Lou Q-Q, Long D-D, Huang J-Y, Zhang Z, Hu S-S, Wang D (2024) Chronic nicotine exposure elicits pain hypersensitivity through activation of dopaminergic projections to anterior cingulate cortex. Br J Anaesth 132:735–745. 10.1016/j.bja.2023.12.034 38336518

[B8] Chen X-F, Guo Y, Lu X-Q, Qi L, Xu K-H, Chen Y, Li G-X, Ding J-P, Li J (2021) Aberrant intraregional brain activity and functional connectivity in patients with diarrhea-predominant irritable bowel syndrome. Front Neurosci 15:721822. 10.3389/fnins.2021.721822 34539337 PMC8446353

[B9] Chen X, et al. (2025) Multimodal abnormalities of brain function in chronic low back pain: a systematic review and meta-analysis of neuroimaging studies. Front Neurosci 19:1535288. 10.3389/fnins.2025.1535288 39975971 PMC11836031

[B10] Cheriyan J, Sheets PL (2018) Altered excitability and local connectivity of mPFC-PAG neurons in a mouse model of neuropathic pain. J Neurosci 38:4829–4839. 10.1523/jneurosci.2731-17.2018 29695413 PMC6596022

[B11] Chiang H-L, Chia Y-Y, Lin H-S, Chen C-H (2016) The implications of tobacco smoking on acute postoperative pain: a prospective observational study. Pain Res Manag 2016:9432493. 10.1155/2016/9432493 27445634 PMC4904603

[B12] Cho YJ, Mehta T, Hinton A, Sloan R, Nshimiyimana J, Tackett AP, Roberts ME, Brinkman MC, Wagener TL (2024) E-Cigarette nicotine delivery among young adults by nicotine form, concentration, and flavor: a crossover randomized clinical trial. JAMA Netw Open 7:e2426702. 10.1001/jamanetworkopen.2024.26702 39120901 PMC11316233

[B13] Cole RD, Zimmerman M, Matchanova A, Kutlu MG, Gould TJ, Parikh V (2020) Cognitive rigidity and BDNF-mediated frontostriatal glutamate neuroadaptations during spontaneous nicotine withdrawal. Neuropsychopharmacology 45:866–876. 10.1038/s41386-019-0574-6 31752015 PMC7075915

[B14] Dai X, Gakidou E, Lopez AD (2022) Evolution of the global smoking epidemic over the past half century: strengthening the evidence base for policy action. Tob Control 31:129–137. 10.1136/tobaccocontrol-2021-056535 35241576

[B15] Ditre JW, Zale EL, LaRowe LR, Kosiba JD, De Vita MJ (2018) Nicotine deprivation increases pain intensity, neurogenic inflammation, and mechanical hyperalgesia among daily tobacco smokers. J Abnorm Psychol 127:578–589. 10.1037/abn0000353 29781659 PMC6089646

[B16] Fang S, et al. (2023) Differences in the neural basis and transcriptomic patterns in acute and persistent pain-related anxiety-like behaviors. Front Mol Neurosci 16:1185243. 10.3389/fnmol.2023.1185243 37383426 PMC10297165

[B17] Fischell SA, Ross TJ, Deng Z-D, Salmeron BJ, Stein EA (2020) Transcranial direct current stimulation applied to the dorsolateral and ventromedial prefrontal cortices in smokers modifies cognitive circuits implicated in the nicotine withdrawal syndrome. Biol Psychiatry Cogn Neurosci Neuroimaging 5:448–460. 10.1016/j.bpsc.2019.12.020 32151567 PMC7150637

[B18] Fransson P, Marrelec G (2008) The precuneus/posterior cingulate cortex plays a pivotal role in the default mode network: evidence from a partial correlation network analysis. Neuroimage 42:1178–1184. 10.1016/j.neuroimage.2008.05.059 18598773

[B19] Garrison KA, Yip SW, Balodis IM, Carroll KM, Potenza MN, Krishnan-Sarin S (2017) Reward-related frontostriatal activity and smoking behavior among adolescents in treatment for smoking cessation. Drug Alcohol Depend 177:268–276. 10.1016/j.drugalcdep.2017.03.035 28651213 PMC5564393

[B20] Goldstein RZ, Volkow ND (2011) Dysfunction of the prefrontal cortex in addiction: neuroimaging findings and clinical implications. Nat Rev Neurosci 12:652–669. 10.1038/nrn3119 22011681 PMC3462342

[B21] Gozen O, Aypar B, Bintepe MO, Tuzcu F, Balkan B, Koylu EO, Kanit L, Keser A (2024) Chronic nicotine consumption and withdrawal regulate melanocortin receptor, CRF, and CRF receptor mRNA levels in the rat brain. Brain Sci 14:63. 10.3390/brainsci14010063 38248278 PMC10813117

[B22] Guo L, Zhang Y, Wang J, Qi Y, Zhang Z (2020) IRF8 is crucial for the nicotine withdrawal-induced hyperalgesia in mice. Transl Neurosci 11:283–293. 10.1515/tnsci-2020-0139 33335768 PMC7712045

[B23] Hooten WM, Townsend CO, Bruce BK, Warner DO (2008) The effects of smoking status on opioid tapering among patients with chronic pain. Anesth Analg 108:308–315. 10.1213/ane.0b013e31818c7b99 19095867

[B24] Howard R, Singh K, Englesbe M (2021) Prevalence and trends in smoking among surgical patients in Michigan, 2012-2019. JAMA Network Open 4:e210553. 10.1001/jamanetworkopen.2021.0553 33656529 PMC7930923

[B25] Hsiao F-J, Wang S-J, Lin Y-Y, Fuh J-L, Ko Y-C, Wang P-N, Chen W-T (2017) Altered insula-default mode network connectivity in fibromyalgia: a resting-state magnetoencephalographic study. J Headache Pain 18:89. 10.1186/s10194-017-0799-x 28831711 PMC5567574

[B26] Huang B, Chen Z, Huang F, Gao F, Chen J, Liu P, Lu Z, Chen W, Wu J (2023) Demyelination in the medial prefrontal cortex by withdrawal from chronic nicotine causes impaired cognitive memory. Prog Neuropsychopharmacol Biol Psychiatry 129:110901. 10.1016/j.pnpbp.2023.110901 38036034

[B27] Huang EY-K, Hung H-Y, Chen Y-H, Kao J-H, Tsai A-L, Chow L-H (2022) Effects of dextromethorphan on nicotine-induced reward, behavioral sensitization, withdrawal signs, and drug seeking-related behavior in rats. Nicotine Tob Res 25:1251–1260. 10.1093/ntr/ntac287 36520961

[B28] Iida H, Yamaguchi S, Goyagi T, Sugiyama Y, Taniguchi C, Matsubara T, Yamada N, Yonekura H, Iida M (2022) Consensus statement on smoking cessation in patients with pain. J Anesth 36:671–687. 10.1007/s00540-022-03097-w 36069935 PMC9666296

[B29] Jin P, Wang F, Zeng F, Yu J, Cui F, Yang B, Zhang L (2024) Revealing the mechanism of central pain hypersensitivity in primary dysmenorrhea: evidence from neuroimaging. Quant Imaging Med Surg 14:3075–3085. 10.21037/qims-23-1687 38617141 PMC11007516

[B30] Kang B, et al. (2023) Electroacupuncture alleviates pain after total knee arthroplasty through regulating neuroplasticity: a resting-state functional magnetic resonance imaging study. Brain Behav 13:e2913. 10.1002/brb3.2913 36749304 PMC10013951

[B31] Keeley RJ, Hsu L-M, Brynildsen JK, Lu H, Yang Y, Stein EA (2020) Intrinsic differences in insular circuits moderate the negative association between nicotine dependence and cingulate-striatal connectivity strength. Neuropsychopharmacology 45:1042–1049. 10.1038/s41386-020-0635-x 32053829 PMC7162949

[B32] Keizer D, van Wijhe M, Post WJ, Wierda JMKH (2007) Quantifying allodynia in patients suffering from unilateral neuropathic pain using von frey monofilaments. Clin J Pain 23:85–90. 10.1097/01.ajp.0000210950.01503.72 17277649

[B33] Kim DH, Park JY, Karm M-H, Bae H-Y, Lee J-Y, Ahn HS, Lee K, Leem JG (2017) Smoking may increase postoperative opioid consumption in patients who underwent distal gastrectomy with gastroduodenostomy for early stomach cancer: a retrospective analysis. Clin J Pain 33:905–911. 10.1097/AJP.0000000000000472 28118255

[B34] Kucyi A, Moayedi M, Weissman-Fogel I, Goldberg MB, Freeman BV, Tenenbaum HC, Davis KD (2014) Enhanced medial prefrontal-default mode network functional connectivity in chronic pain and its association with pain rumination. J Neurosci 34:3969–3975. 10.1523/jneurosci.5055-13.2014 24623774 PMC6705280

[B35] Lai P, et al. (2025) Abnormalities of insular functional connectivity in patients with musculoskeletal pain: a meta-analysis of resting-state fMRI studies. Brain Res Bull 224:111294. 10.1016/j.brainresbull.2025.111294 40081505

[B36] Lan L, et al. (2022) Acupuncture modulates the spontaneous activity and functional connectivity of calcarine in patients with chronic stable angina pectoris. Front Mol Neurosci 15:842674. 10.3389/fnmol.2022.842674 35557556 PMC9087858

[B37] LaRowe LR, Ditre JW (2020) Pain, nicotine, and tobacco smoking: current state of the science. Pain 161:1688–1693. 10.1097/j.pain.0000000000001874 32701828 PMC8924914

[B38] Lee YC, Nassikas NJ, Clauw DJ (2011) The role of the central nervous system in the generation and maintenance of chronic pain in rheumatoid arthritis, osteoarthritis and fibromyalgia. Arthritis Res Ther 13:211. 10.1186/ar3306 21542893 PMC3132050

[B39] Lesage E, Sutherland MT, Ross TJ, Salmeron BJ, Stein EA (2020) Nicotine dependence (trait) and acute nicotinic stimulation (state) modulate attention but not inhibitory control: converging fMRI evidence from Go-Nogo and Flanker tasks. Neuropsychopharmacology 45:857–865. 10.1038/s41386-020-0623-1 31995811 PMC7075893

[B40] Lin W, Zhou F, Yu L, Wan L, Yuan H, Wang K, Svensson P (2020) Quantitative sensory testing of periauricular skin in healthy adults. Sci Rep 10:3728. 10.1038/s41598-020-60724-w 32111937 PMC7048815

[B41] Loeser JD, Melzack R (1999) Pain: an overview. Lancet 353:1607–1609. 10.1016/s0140-6736(99)01311-2 10334273

[B42] Long D-D, Zhang Y-Z, Liu A, Shen L, Wei H-R, Lou Q-Q, Hu S-S, Chen D-Y, Chai X-Q, Wang D (2023) Microglia sustain anterior cingulate cortex neuronal hyperactivity in nicotine-induced pain. J Neuroinflammation 20:81. 10.1186/s12974-023-02767-0 36944965 PMC10031886

[B43] Lv S-S, Lv X-J, Cai Y-Q, Hou X-Y, Zhang Z-Z, Wang G-H, Chen L-Q, Lv N, Zhang Y-Q (2024) Corticotropin-releasing hormone neurons control trigeminal neuralgia-induced anxiodepression via a hippocampus-to-prefrontal circuit. Sci Adv 10:eadj4196. 10.1126/sciadv.adj4196 38241377 PMC10798562

[B44] Ma L, Tao Q, Dang J, Sun J, Niu X, Zhang M, Kang Y, Wang W, Cheng J, Zhang Y (2025) The structural and functional brain alternations in tobacco use disorder: a systematic review and meta-analysis. Front Psychiatry 16:1403604. 10.3389/fpsyt.2025.1403604 40291519 PMC12022757

[B45] Maulitz L, Nehls S, Stickeler E, Ignatov A, Kupec T, Henn AT, Chechko N, Tchaikovski SN (2024) Psychological characteristics and structural brain changes in women with endometriosis and endometriosis-independent chronic pelvic pain. Hum Reprod 39:2473–2484. 10.1093/humrep/deae207 39241806

[B46] Menossi HS, Goudriaan AE, de Azevedo-Marques Périco C, Nicastri S, de Andrade AG, D'Elia G, Li C-SR, Castaldelli-Maia JM (2013) Neural bases of pharmacological treatment of nicotine dependence - insights from functional brain imaging: a systematic review. CNS Drugs 27:921–941. 10.1007/s40263-013-0092-8 23853032

[B47] Mosch B, Hagena V, Herpertz S, Ruttorf M, Diers M (2023) Neural correlates of control over pain in fibromyalgia patients. Neuroimage Clin 37:103355. 10.1016/j.nicl.2023.103355 36848728 PMC9982683

[B48] Ong W-Y, Stohler CS, Herr DR (2019) Role of the prefrontal cortex in pain processing. Mol Neurobiol 56:1137–1166. 10.1007/s12035-018-1130-9 29876878 PMC6400876

[B49] Ou Y, et al. (2024) Structural and functional changes of anterior cingulate cortex subregions in migraine without aura: relationships with pain sensation and pain emotion. Cereb Cortex 34:bhae040. 10.1093/cercor/bhae040 38342690 PMC10859245

[B50] Pan Q, Guo S-S, Chen M, Su X-Y, Gao Z-L, Wang Q, Xu T-L, Liu M-G, Hu J (2023) Representation and control of pain and itch by distinct prefrontal neural ensembles. Neuron 111:2414–2431.e7. 10.1016/j.neuron.2023.04.032 37224813

[B51] Qiu D, et al. (2023) Brain structure and cortical activity changes of new daily persistent headache: multimodal evidence from MEG/sMRI. J Headache Pain 24:45. 10.1186/s10194-023-01581-6 37098498 PMC10129440

[B52] Schmidt BL, Tambeli CH, Gear RW, Levine JD (2001) Nicotine withdrawal hyperalgesia and opioid-mediated analgesia depend on nicotine receptors in nucleus accumbens. Neuroscience 106:129–136. 10.1016/s0306-4522(01)00264-0 11564423

[B53] Shao J, Fei Y, Xiao J, Wang L, Zou S, Yang J (2023) The role of miRNA-144-3p/Oprk1/KOR in nicotine dependence and nicotine withdrawal in male rats. Nicotine Tob Res 25:1856–1864. 10.1093/ntr/ntad118 37455648 PMC10664084

[B54] Shen L, et al. (2017) Decreased pain tolerance before surgery and increased postoperative narcotic requirements in abstinent tobacco smokers. Addict Behav 78:9–14. 10.1016/j.addbeh.2017.10.024 29121531

[B55] Shen L, Qiu H-B, Xu H-H, Wei K, Zhao L, Zhu C-C, Li C-J, Lu Z-J (2021) Nicotine withdrawal induces hyperalgesia via downregulation of descending serotonergic pathway in the nucleus raphe magnus. Neuropharmacology 189:108515. 10.1016/j.neuropharm.2021.108515 33722649

[B56] Shepherd J, Li S, Herring E, Labak CM, Miller JP (2024) Tobacco use and trigeminal neuralgia: clinical features and outcome after microvascular decompression. Neurosurgery 96:667–672. 10.1227/neu.0000000000003192 39324787

[B57] Shirvalkar P, et al. (2023) First-in-human prediction of chronic pain state using intracranial neural biomarkers. Nat Neurosci 26:1090–1099. 10.1038/s41593-023-01338-z 37217725 PMC10330878

[B58] Sussman S, Dent CW, Nezami E, Stacy AW, Burton D, Flay BR (1998) Reasons for quitting and smoking temptation among adolescent smokers: gender differences. Subst Use Misuse 33:2703–2720. 10.3109/10826089809059346 9869439

[B59] Sutherland MT, Ray KL, Riedel MC, Yanes JA, Stein EA, Laird AR (2015) Neurobiological impact of nicotinic acetylcholine receptor agonists: an activation likelihood estimation meta-analysis of pharmacologic neuroimaging studies. Biol Psychiatry 78:711–720. 10.1016/j.biopsych.2014.12.021 25662104 PMC4494985

[B60] Sutherland MT, Riedel MC, Flannery JS, Yanes JA, Fox PT, Stein EA, Laird AR (2016) Chronic cigarette smoking is linked with structural alterations in brain regions showing acute nicotinic drug-induced functional modulations. Behav Brain Funct 12:16. 10.1186/s12993-016-0100-5 27251183 PMC4890474

[B61] Sutherland MT, Stein EA (2018) Functional neurocircuits and neuroimaging biomarkers of tobacco use disorder. Trends Mol Med 24:129–143. 10.1016/j.molmed.2017.12.002 29398401 PMC5928775

[B62] Sweitzer MM, Geier CF, Addicott MA, Denlinger R, Raiff BR, Dallery J, McClernon FJ, Donny EC (2016) Smoking abstinence-induced changes in resting state functional connectivity with ventral striatum predict lapse during a quit attempt. Neuropsychopharmacology 41:2521–2529. 10.1038/npp.2016.56 27091382 PMC4987851

[B63] Wang C, Zhang Y, Yan C, Sun M, Cheng J (2018) The thalamo-cortical resting state functional connectivity and abstinence-induced craving in young smokers. Brain Imaging Behav 12:1450–1456. 10.1007/s11682-017-9809-5 29297152

[B64] Wang T, Li R, Chen D, Xie M, Li Z, Mao H, Ling Y, Liang X, Xu G, Zhang J (2024) Modulation of high-frequency rTMS on reward circuitry in individuals with nicotine dependence: a preliminary fMRI study. Neural Plast 2024:5673579. 10.1155/2024/5673579 39234068 PMC11374416

[B65] Wei B, Feng J, Rehmani IJ, Miller S, McGuffey JE, Blount BC, Wang L (2014) A high-throughput robotic sample preparation system and HPLC-MS/MS for measuring urinary anatabine, anabasine, nicotine and major nicotine metabolites. Clin Chim Acta 436:290–297. 10.1016/j.cca.2014.06.012 24968308 PMC4516125

[B66] Wei K, Bi Y, Xu H, Shen L, Liu Y, Chen Q, Miao X, Wu C, Hu L, Lu Z-J (2023) The effect of tramadol versus sufentanil on controlling postoperative pain for men who smoke and do not smoke: a randomized clinical trial. Pain Physician 25:E1367–E1377. 36608008

[B67] Weingarten TN, Sprung J, Flores A, Baena AMO, Schroeder DR, Warner DO (2011) Opioid requirements after laparoscopic bariatric surgery. Obes Surg 21:1407–1412. 10.1007/s11695-010-0217-9 20563662

[B68] Wen Z, Han X, Wang Y, Ding W, Sun Y, Kang Y, Zhou Y, Lei H, Lin F (2022) Sex-dependent alterations of regional homogeneity in cigarette smokers. Front Psychiatry 13:874893. 10.3389/fpsyt.2022.874893 35546937 PMC9082268

[B69] Wu G, Luo Y, Guo D, Lv S, Yang J (2025) A meta-analysis of resting-state fMRI in postherpetic neuralgia using AES-SDM. Front Neurosci 19:1556639. 10.3389/fnins.2025.1556639 40236945 PMC11998669

[B70] Yague JG, Foffani G, Aguilar J (2010) Cortical hyperexcitability in response to preserved spinothalamic inputs immediately after spinal cord hemisection. Exp Neurol 227:252–263. 10.1016/j.expneurol.2010.11.011 21093438

[B71] Yan C-G, Wang X-D, Zuo X-N, Zang Y-F (2016) DPABI: data processing & analysis for (resting-state) brain imaging. Neuroinformatics 14:339–351. 10.1007/s12021-016-9299-4 27075850

[B72] Yarnitsky D, Granot M, Granovsky Y (2013) Pain modulation profile and pain therapy: between pro- and antinociception. Pain 155:663–665. 10.1016/j.pain.2013.11.005 24269491

[B73] Yip SW, Lichenstein SD, Garrison K, Averill CL, Viswanath H, Salas R, Abdallah CG (2022) Effects of smoking status and state on intrinsic connectivity. Biol Psychiatry Cogn Neurosci Neuroimaging 7:895–904. 10.1016/j.bpsc.2021.02.004 33618016 PMC8373998

[B74] Yu Z, Wang R-R, Wei W, Liu L-Y, Wen C-B, Yu S-G, Guo X-L, Yang J (2023) A coordinate-based meta-analysis of acupuncture for chronic pain: evidence from fMRI studies. Front Neurosci 16:1049887. 10.3389/fnins.2022.1049887 PMC9795831 36590302

[B75] Yuan Z, et al. (2022) Altered functional connectivity of the right caudate nucleus in chronic migraine: a resting-state fMRI study. J Headache Pain 23:154. 10.1186/s10194-022-01506-9 36460958 PMC9717534

[B76] Zhang M, Dang J, Sun J, Tao Q, Niu X, Wang W, Han S, Cheng J, Zhang Y (2024) Effective connectivity of default mode network subsystems and automatic smoking behaviour among males. J Psychiatry Neurosci 49:E429–E439. 10.1503/jpn.240058 39689937 PMC11665814

[B77] Zhang R, Volkow ND (2019) Brain default-mode network dysfunction in addiction. Neuroimage 200:313–331. 10.1016/j.neuroimage.2019.06.036 31229660

[B78] Zhang Y, Yang J, Sevilla A, Weller R, Wu J, Su C, Zheng C, Rodriguez-Blanco YF, Gitlin M, Candiotti KA (2019) The mechanism of chronic nicotine exposure and nicotine withdrawal on pain perception in an animal model. Neurosci Lett 715:134627. 10.1016/j.neulet.2019.134627 31733321

[B79] Zhao S, Li Y, Li M, Wang R, Bi Y, Zhang Y, Lu X, Yu D, Yang L, Yuan K (2018) 12-H abstinence-induced functional connectivity density changes and craving in young smokers: a resting-state study. Brain Imaging Behav 13:953–962. 10.1007/s11682-018-9911-3 29926324

[B80] Zhu C, Bi Y, Wei K, Tao K, Hu L, Lu Z (2023) Effect of perioperative high-dose transdermal nicotine patch on pain sensitivity among male abstinent tobacco smokers undergoing abdominal surgery: a randomized controlled pilot study. Addiction 118:1579–1585. 10.1111/add.16224 37132069

[B81] Zhu J, Gu R, Shi L, Su Y (2023) Altered intrinsic brain activity in patients with neuropathic pain after brachial plexus avulsion. Brain Res Bull 206:110831. 10.1016/j.brainresbull.2023.110831 38056510

[B82] Zong X, et al. (2019) A dissociation in effects of risperidone monotherapy on functional and anatomical connectivity within the default mode network. Schizophr Bull 45:1309–1318. 10.1093/schbul/sby175 30508134 PMC6811838

